# 4-(4-Cyano-2-fluoro­phen­oxy)phenyl 4-methyl­benzene­sulfonate

**DOI:** 10.1107/S1600536809029201

**Published:** 2009-07-29

**Authors:** Shuping Luo, Jixu Zhang, Jianfeng Wang, Bailin Li

**Affiliations:** a State Key Laboratory Breeding Base of Green Chemistry-Synthesis Technology, Zhejiang University of Technology, Hangzhou 310014, People’s Republic of China

## Abstract

The title compound, C_20_H_14_FNO_4_S, was synthesized from hydro­quinone, *p*-toluene­sulfonyl chloride and 3,4-difluoro­benzonitrile. A folded conformation is adopted by the crystal structure. Inter­molecular C—H⋯N hydrogen bonds form dimers arranged around inversion centers.

## Related literature

For the herbicidal activity of hydro­quinone derivatives, see: Bao *et al.* (2007 ▶); Liu (2002 ▶). For related structures, see: Chen & Zhang (2009 ▶); Han *et al.* (2008 ▶); Yang *et al.* (2008 ▶). For hydrogen-bond motifs, see: Bernstein *et al.* (1995 ▶); Etter (1990 ▶).
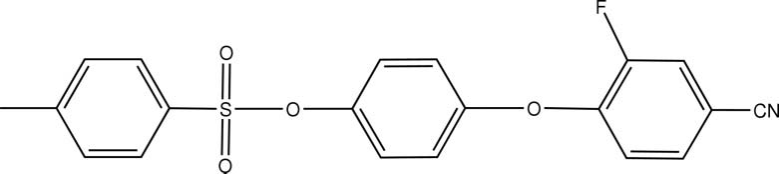


## Experimental

### 

#### Crystal data

C_20_H_14_FNO_4_S
*M* *_r_* = 383.39
Triclinic, 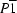

*a* = 7.5504 (4) Å
*b* = 9.9558 (6) Å
*c* = 12.5862 (6) Å
α = 89.5250 (15)°
β = 77.8080 (12)°
γ = 81.9370 (15)°
*V* = 915.40 (9) Å^3^
*Z* = 2
Mo *K*α radiation
μ = 0.21 mm^−1^
*T* = 296 K
0.42 × 0.32 × 0.28 mm

#### Data collection

Rigaku R-AXIS RAPID diffractometer
Absorption correction: multi-scan (*ABSCOR*; Higashi, 1995 ▶) *T*_min_ = 0.910, *T*_max_ = 0.942
9012 measured reflections
4114 independent reflections
2386 reflections with *F* ^2^ > 2σ(*F* ^2^)
*R*_int_ = 0.025

#### Refinement

*R*[*F* ^2^ > 2σ(*F* ^2^)] = 0.041
*wR*(*F* ^2^) = 0.123
*S* = 1.01
4114 reflections
245 parameters
H-atom parameters constrained
Δρ_max_ = 0.46 e Å^−3^
Δρ_min_ = −0.50 e Å^−3^

### 

Data collection: *PROCESS-AUTO* (Rigaku, 1998 ▶); cell refinement: *PROCESS-AUTO*; data reduction: *CrystalStructure* (Rigaku/MSC, 2004 ▶), and Larson (1970 ▶); program(s) used to solve structure: *SHELXS97* (Sheldrick, 2008 ▶); program(s) used to refine structure: *CRYSTALS* (Betteridge *et al.*, 2003 ▶); molecular graphics: *ORTEP-3 for Windows* (Farrugia, 1997 ▶) and *PLATON* (Spek, 2009 ▶); software used to prepare material for publication: *CrystalStructure*.

## Supplementary Material

Crystal structure: contains datablocks global, I. DOI: 10.1107/S1600536809029201/dn2473sup1.cif

Structure factors: contains datablocks I. DOI: 10.1107/S1600536809029201/dn2473Isup2.hkl

Additional supplementary materials:  crystallographic information; 3D view; checkCIF report

## Figures and Tables

**Table 1:** Hydrogen-bond geometry (Å, °)

*D*—H⋯*A*	*D*—H	H⋯*A*	*D*⋯*A*	*D*—H⋯*A*
C16—H16⋯N1^i^	0.93	2.61	3.461 (3)	152

Symmetry code: (i) 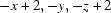.

**Fig. 1.**
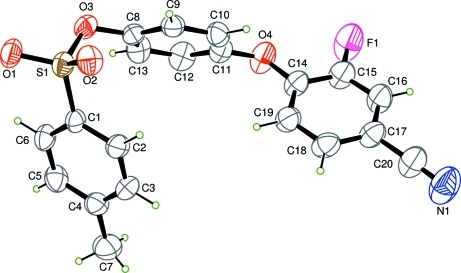
Molecular structure of title compound, with the atomic labeling scheme. Displacement ellipsoids are drawn at the 50% probability level.

**Fig. 2.**
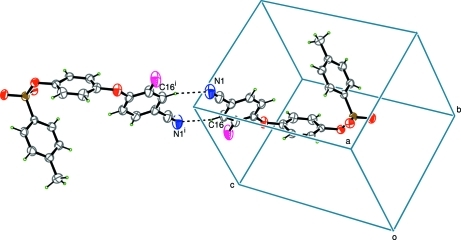
A partial packing diagram of title compound. Hydrogen bonds are shown as dashed lines. [Symmetry code: (i) -x+2, -y, -z+2].

## supplementary crystallographic information

### Comment

The herbicidal activity of hydroquinone derivatives is well known in the art (Liu, 2002. Bao *et al.*, 2007). As part of our ongoing studies, we now describe the synthesis and the crystal structure of the title compound.

As shown in Fig.1, the terminal C1—C7/S1 phenyl ring, the central benzene ring (C8—C13/O3/O4) and the other terminal phenyl ring (C14—C20/N1) form three planes, with max deviations for fitted atoms of 0.042 Å, 0.022Å and 0.013 Å, respectively. These planes make dihedral angles of 45.0 (1)° and 64.6 (6)° respectively. Otherwise, the molecule is bent at the sulfonate group with the C1—S1—O3—C8 torsion angle of 50.7 (3). The other bond parameters are similar to those observed in 4-Methyl-2-oxo-2,3-dihydro-1-benzopyran-7-yl benzenesulfonate (Yang *et al.*, 2008), (*E*)-4-[(1,5-Dimethyl-3-oxo-2-phenyl-2,3-dihydro-1*H*-pyrazol-4-yl) iminomethyl]phenyl 4-bromobenzenesulfonate (Han *et al.*, 2008) and 2-Methyl-3-nitrobenzyl cyanide (Chen *et al.*, 2009).

In the crystal structure, the molecules are linked to form pseudo dimers by inter molecular C—H···N hydrogen bonds generating a graph set motif *R*_2_^2^(10) (Table 1, Fig.2) (Etter, 1990, Bernstein *et al.*,  1995). In addition, the structure is stabilized by weak C—H···O and van der Waal's interactions.

### Experimental

A DMSO (10 ml) solution of hydroquinone and *p*-toluenesulfonyl chloride in the presence of KOH as base was stirred at room temperature for 48 h. Then the mixture was heated to 70°C and 3,4-difluorobenzonitrile was added dropwise. Finally the mixture was washed with water (20 ml) and extracted with ethyl acetate (three times). The organic solvent was removed under reduced pressure and the product was purified by silica gel chromatography (pentane: ethyl acetate mixtures). Suitable crystals were obtained by slow evaporation of ethanol at room temperature.

### Refinement

All H atoms were placed in calculated positions with C—H = 0.93–0.98 Å, and were included in the refinement in the riding model with *U*_iso_(H) = 1.2*U*_eq_ of the carrier atoms.

### Crystal data

**Table:**

C_20_H_14_FNO_4_S	*Z* = 2
*M**_r_* = 383.39	*F*(000) = 396.00
Triclinic, *P*1	*D*_x_ = 1.391 Mg m^−^^3^
Hall symbol: -P 1	Mo *K*α radiation, λ = 0.71075 Å
*a* = 7.5504 (4) Å	Cell parameters from 5883 reflections
*b* = 9.9558 (6) Å	θ = 3.2–27.4°
*c* = 12.5862 (6) Å	µ = 0.21 mm^−^^1^
α = 89.5250 (15)°	*T* = 296 K
β = 77.8080 (12)°	Chunk, colorless
γ = 81.9370 (15)°	0.42 × 0.32 × 0.28 mm
*V* = 915.40 (9) Å^3^	

### Data collection

**Table:**

Rigaku R-AXIS RAPID diffractometer	2386 reflections with *F*^2^ > 2σ(*F*^2^)
Detector resolution: 10.00 pixels mm^-1^	*R*_int_ = 0.025
ω scans	θ_max_ = 27.4°
Absorption correction: multi-scan (*ABSCOR*; Higashi, 1995)	*h* = −9→9
*T*_min_ = 0.910, *T*_max_ = 0.942	*k* = −12→12
9012 measured reflections	*l* = −16→16
4114 independent reflections	

### Refinement

**Table:**

Refinement on *F*^2^	*w* = 1/[0.0006*F*_o_^2^ + 2σ(*F*_o_^2^)]/(4*F*_o_^2^)
*R*[*F*^2^ > 2σ(*F*^2^)] = 0.041	(Δ/σ)_max_ < 0.001
*wR*(*F*^2^) = 0.123	Δρ_max_ = 0.46 e Å^−^^3^
*S* = 1.01	Δρ_min_ = −0.50 e Å^−^^3^
4114 reflections	Extinction correction: Larson (1970)
245 parameters	Extinction coefficient: 591 (29)
H-atom parameters constrained	

### Special details

**Table:**

Geometry. ENTER SPECIAL DETAILS OF THE MOLECULAR GEOMETRY
Refinement. Refinement using all reflections. The weighted *R*-factor (*wR*) and goodness of fit (*S*) are based on *F*^2^. *R*-factor (gt) are based on *F*. The threshold expression of *F*^2^ > 2.0 σ(*F*^2^) is used only for calculating *R*-factor (gt).

### Fractional atomic coordinates and isotropic or equivalent isotropic displacement parameters (Å^2^)

**Table:**

	*x*	*y*	*z*	*U*_iso_*/*U*_eq_	
S1	0.16109 (6)	0.69907 (6)	0.48722 (4)	0.05510 (18)	
F1	0.3393 (2)	0.2172 (2)	1.07043 (12)	0.1276 (7)	
O1	0.0493 (2)	0.78677 (14)	0.43051 (12)	0.0702 (5)	
O2	0.2714 (2)	0.58197 (13)	0.43277 (12)	0.0653 (4)	
O3	0.01598 (18)	0.65207 (16)	0.58710 (12)	0.0606 (4)	
O4	0.2671 (2)	0.4109 (2)	0.93536 (12)	0.0767 (6)	
N1	1.1212 (3)	0.0868 (3)	0.8909 (2)	0.1196 (11)	
C1	0.2945 (2)	0.7893 (2)	0.54964 (16)	0.0488 (6)	
C2	0.4542 (2)	0.7240 (2)	0.57449 (19)	0.0607 (7)	
C3	0.5538 (2)	0.7947 (2)	0.6284 (2)	0.0670 (8)	
C4	0.4972 (3)	0.9299 (2)	0.65809 (18)	0.0635 (7)	
C5	0.3378 (3)	0.9927 (2)	0.63241 (19)	0.0680 (7)	
C6	0.2358 (3)	0.9238 (2)	0.57855 (18)	0.0604 (7)	
C7	0.6053 (4)	1.0073 (3)	0.7184 (2)	0.0936 (10)	
C8	0.0820 (2)	0.5862 (2)	0.67430 (18)	0.0522 (6)	
C9	0.1745 (2)	0.4561 (2)	0.66080 (18)	0.0554 (6)	
C10	0.2391 (2)	0.3966 (2)	0.74779 (18)	0.0613 (7)	
C11	0.2089 (2)	0.4676 (2)	0.84456 (18)	0.0614 (7)	
C12	0.1107 (3)	0.5944 (2)	0.8574 (2)	0.0739 (8)	
C13	0.0470 (3)	0.6553 (2)	0.7708 (2)	0.0704 (8)	
C14	0.4443 (3)	0.3479 (2)	0.92161 (17)	0.0645 (7)	
C15	0.4799 (3)	0.2471 (3)	0.99270 (19)	0.0762 (8)	
C16	0.6506 (3)	0.1772 (2)	0.98788 (19)	0.0780 (8)	
C17	0.7936 (3)	0.2105 (2)	0.90833 (18)	0.0687 (8)	
C18	0.7627 (3)	0.3131 (2)	0.8374 (2)	0.0742 (8)	
C19	0.5882 (3)	0.3820 (2)	0.84441 (19)	0.0716 (8)	
C20	0.9752 (3)	0.1401 (3)	0.8989 (2)	0.0856 (10)	
H2	0.4940	0.6332	0.5550	0.073*	
H3	0.6611	0.7507	0.6452	0.080*	
H5	0.2980	1.0836	0.6518	0.082*	
H6	0.1283	0.9677	0.5619	0.072*	
H9	0.1932	0.4093	0.5949	0.066*	
H10	0.3026	0.3090	0.7407	0.074*	
H12	0.0867	0.6399	0.9243	0.089*	
H13	−0.0188	0.7421	0.7786	0.085*	
H16	0.6703	0.1090	1.0368	0.094*	
H18	0.8594	0.3360	0.7847	0.089*	
H19	0.5681	0.4516	0.7968	0.086*	
H71	0.5630	0.9986	0.7953	0.112*	
H72	0.5889	1.1014	0.7001	0.112*	
H73	0.7328	0.9711	0.6981	0.112*	

### Atomic displacement parameters (Å^2^)

**Table:**

	*U*^11^	*U*^22^	*U*^33^	*U*^12^	*U*^13^	*U*^23^
S1	0.0528 (3)	0.0515 (3)	0.0600 (3)	0.0018 (2)	−0.0156 (2)	0.0017 (2)
F1	0.0771 (10)	0.191 (2)	0.0934 (11)	0.0040 (11)	0.0113 (9)	0.0689 (12)
O1	0.0738 (10)	0.0636 (11)	0.0790 (10)	0.0040 (8)	−0.0384 (9)	0.0084 (8)
O2	0.0729 (10)	0.0498 (10)	0.0666 (9)	0.0062 (8)	−0.0093 (8)	−0.0106 (7)
O3	0.0409 (7)	0.0681 (10)	0.0715 (9)	−0.0008 (7)	−0.0141 (7)	0.0079 (8)
O4	0.0641 (10)	0.1006 (16)	0.0548 (9)	0.0083 (10)	−0.0018 (7)	0.0114 (9)
N1	0.0747 (17)	0.165 (2)	0.1032 (19)	0.0130 (18)	−0.0039 (14)	0.0380 (19)
C1	0.0450 (11)	0.0443 (13)	0.0541 (11)	0.0034 (9)	−0.0102 (9)	0.0015 (9)
C2	0.0450 (12)	0.0501 (14)	0.0829 (15)	0.0025 (11)	−0.0104 (11)	−0.0006 (12)
C3	0.0465 (12)	0.0709 (18)	0.0860 (17)	−0.0043 (12)	−0.0224 (12)	0.0092 (14)
C4	0.0649 (15)	0.0703 (18)	0.0600 (13)	−0.0209 (13)	−0.0165 (11)	0.0107 (12)
C5	0.0849 (17)	0.0489 (15)	0.0714 (15)	−0.0026 (13)	−0.0239 (13)	−0.0035 (12)
C6	0.0632 (14)	0.0500 (15)	0.0671 (14)	0.0072 (12)	−0.0217 (11)	−0.0003 (11)
C7	0.104 (2)	0.102 (2)	0.0925 (19)	−0.0405 (19)	−0.0434 (17)	0.0119 (17)
C8	0.0375 (10)	0.0554 (14)	0.0617 (13)	−0.0048 (10)	−0.0070 (9)	0.0044 (11)
C9	0.0535 (12)	0.0537 (14)	0.0606 (13)	−0.0104 (11)	−0.0140 (10)	−0.0015 (11)
C10	0.0568 (13)	0.0537 (15)	0.0693 (15)	−0.0022 (11)	−0.0078 (11)	0.0036 (12)
C11	0.0494 (13)	0.0771 (18)	0.0532 (13)	−0.0028 (12)	−0.0047 (10)	0.0063 (12)
C12	0.0739 (16)	0.082 (2)	0.0560 (14)	0.0051 (15)	−0.0019 (12)	−0.0138 (13)
C13	0.0649 (15)	0.0649 (17)	0.0692 (15)	0.0126 (13)	−0.0006 (12)	−0.0062 (13)
C14	0.0558 (14)	0.0872 (19)	0.0487 (12)	−0.0056 (13)	−0.0100 (11)	0.0058 (12)
C15	0.0596 (15)	0.109 (2)	0.0517 (13)	−0.0074 (15)	0.0026 (12)	0.0185 (14)
C16	0.0692 (16)	0.100 (2)	0.0597 (14)	−0.0042 (15)	−0.0077 (13)	0.0218 (14)
C17	0.0581 (14)	0.091 (2)	0.0558 (13)	−0.0062 (13)	−0.0126 (11)	0.0020 (13)
C18	0.0587 (15)	0.106 (2)	0.0598 (14)	−0.0209 (14)	−0.0108 (11)	0.0124 (14)
C19	0.0630 (15)	0.092 (2)	0.0617 (14)	−0.0169 (14)	−0.0156 (12)	0.0184 (13)
C20	0.0653 (17)	0.119 (2)	0.0667 (16)	0.0003 (17)	−0.0093 (14)	0.0178 (16)

### Geometric parameters (Å, °)

**Table:**

S1—O1	1.4220 (16)	C14—C15	1.376 (3)
S1—O2	1.4221 (13)	C14—C19	1.375 (3)
S1—O3	1.5975 (14)	C15—C16	1.366 (3)
S1—C1	1.744 (2)	C16—C17	1.384 (3)
F1—C15	1.348 (2)	C17—C18	1.379 (3)
O3—C8	1.418 (2)	C17—C20	1.431 (3)
O4—C11	1.397 (2)	C18—C19	1.383 (3)
O4—C14	1.371 (2)	C2—H2	0.930
N1—C20	1.139 (3)	C3—H3	0.930
C1—C2	1.382 (2)	C5—H5	0.930
C1—C6	1.377 (3)	C6—H6	0.930
C2—C3	1.375 (3)	C7—H71	0.960
C3—C4	1.384 (3)	C7—H72	0.960
C4—C5	1.376 (3)	C7—H73	0.960
C4—C7	1.511 (4)	C9—H9	0.930
C5—C6	1.378 (3)	C10—H10	0.930
C8—C9	1.376 (3)	C12—H12	0.930
C8—C13	1.359 (3)	C13—H13	0.930
C9—C10	1.386 (3)	C16—H16	0.930
C10—C11	1.374 (3)	C18—H18	0.930
C11—C12	1.363 (3)	C19—H19	0.930
C12—C13	1.384 (3)		
			
O1—S1—O2	119.42 (9)	C16—C17—C18	120.1 (2)
O1—S1—O3	102.89 (8)	C16—C17—C20	120.8 (2)
O1—S1—C1	111.32 (10)	C18—C17—C20	119.1 (2)
O2—S1—O3	108.85 (8)	C17—C18—C19	120.2 (2)
O2—S1—C1	109.48 (9)	C14—C19—C18	120.2 (2)
O3—S1—C1	103.47 (8)	N1—C20—C17	178.4 (3)
S1—O3—C8	118.35 (12)	C1—C2—H2	120.3
C11—O4—C14	118.07 (15)	C3—C2—H2	120.3
S1—C1—C2	119.91 (17)	C2—C3—H3	119.4
S1—C1—C6	119.74 (16)	C4—C3—H3	119.4
C2—C1—C6	120.3 (2)	C4—C5—H5	119.3
C1—C2—C3	119.4 (2)	C6—C5—H5	119.3
C2—C3—C4	121.2 (2)	C1—C6—H6	120.3
C3—C4—C5	118.3 (2)	C5—C6—H6	120.3
C3—C4—C7	121.3 (2)	C4—C7—H71	109.5
C5—C4—C7	120.4 (2)	C4—C7—H72	109.5
C4—C5—C6	121.4 (2)	C4—C7—H73	109.5
C1—C6—C5	119.4 (2)	H71—C7—H72	109.5
O3—C8—C9	120.46 (19)	H71—C7—H73	109.5
O3—C8—C13	117.51 (19)	H72—C7—H73	109.5
C9—C8—C13	122.0 (2)	C8—C9—H9	120.8
C8—C9—C10	118.4 (2)	C10—C9—H9	120.8
C9—C10—C11	119.7 (2)	C9—C10—H10	120.1
O4—C11—C10	121.9 (2)	C11—C10—H10	120.1
O4—C11—C12	117.2 (2)	C11—C12—H12	120.1
C10—C11—C12	120.8 (2)	C13—C12—H12	120.1
C11—C12—C13	119.9 (2)	C8—C13—H13	120.5
C8—C13—C12	119.0 (2)	C12—C13—H13	120.5
O4—C14—C15	117.19 (19)	C15—C16—H16	120.9
O4—C14—C19	124.5 (2)	C17—C16—H16	120.9
C15—C14—C19	118.3 (2)	C17—C18—H18	119.9
F1—C15—C14	118.0 (2)	C19—C18—H18	119.9
F1—C15—C16	119.1 (2)	C14—C19—H19	119.9
C14—C15—C16	122.9 (2)	C18—C19—H19	119.9
C15—C16—C17	118.3 (2)		
			
O1—S1—O3—C8	−166.73 (15)	C4—C5—C6—C1	−0.1 (2)
O1—S1—C1—C2	−156.85 (16)	O3—C8—C9—C10	178.99 (19)
O1—S1—C1—C6	26.20 (19)	O3—C8—C13—C12	−179.5 (2)
O2—S1—O3—C8	65.64 (17)	C9—C8—C13—C12	1.9 (3)
O2—S1—C1—C2	−22.63 (19)	C13—C8—C9—C10	−2.4 (3)
O2—S1—C1—C6	160.42 (16)	C8—C9—C10—C11	0.3 (3)
O3—S1—C1—C2	93.29 (17)	C9—C10—C11—O4	178.3 (2)
O3—S1—C1—C6	−83.66 (17)	C9—C10—C11—C12	2.2 (3)
C1—S1—O3—C8	−50.73 (17)	O4—C11—C12—C13	−179.1 (2)
S1—O3—C8—C9	−71.2 (2)	C10—C11—C12—C13	−2.8 (3)
S1—O3—C8—C13	110.10 (19)	C11—C12—C13—C8	0.8 (3)
C11—O4—C14—C15	−153.2 (2)	O4—C14—C15—F1	0.0 (3)
C11—O4—C14—C19	29.1 (3)	O4—C14—C15—C16	−179.7 (2)
C14—O4—C11—C10	48.8 (3)	O4—C14—C19—C18	179.5 (2)
C14—O4—C11—C12	−134.9 (2)	C15—C14—C19—C18	1.8 (4)
S1—C1—C2—C3	−176.91 (16)	C19—C14—C15—F1	177.9 (2)
S1—C1—C6—C5	176.99 (16)	C19—C14—C15—C16	−1.9 (4)
C2—C1—C6—C5	0.1 (2)	F1—C15—C16—C17	−179.2 (2)
C6—C1—C2—C3	0.0 (2)	C14—C15—C16—C17	0.5 (4)
C1—C2—C3—C4	−0.1 (2)	C15—C16—C17—C18	0.9 (4)
C2—C3—C4—C5	0.0 (2)	C15—C16—C17—C20	−179.6 (2)
C2—C3—C4—C7	179.3 (2)	C16—C17—C18—C19	−0.9 (4)
C3—C4—C5—C6	0.0 (2)	C20—C17—C18—C19	179.6 (2)
C7—C4—C5—C6	−179.3 (2)	C17—C18—C19—C14	−0.5 (4)

### Hydrogen-bond geometry (Å, °)

**Table:**

*D*—H···*A*	*D*—H	H···*A*	*D*···*A*	*D*—H···*A*
C16—H16···N1^i^	0.93	2.61	3.461 (3)	152

Symmetry codes: (i) −*x*+2, −*y*, −*z*+2.
